# Base-displaced intercalation of the 2-amino-3-methylimidazo[4,5-*f*]quinolone *N*^2^-dG adduct in the *Nar*I DNA recognition sequence

**DOI:** 10.1093/nar/gkt1109

**Published:** 2013-12-22

**Authors:** Kallie M. Stavros, Edward K. Hawkins, Carmelo J. Rizzo, Michael P. Stone

**Affiliations:** Department of Chemistry, Center in Molecular Toxicology, Vanderbilt-Ingram Cancer Center, Vanderbilt Institute of Chemical Biology, Vanderbilt University, Nashville, TN 37235-1822, USA

## Abstract

2-Amino-3-methylimidazo[4,5-*f*]quinolone (IQ), a heterocyclic amine found in cooked meats, undergoes bioactivation to a nitrenium ion, which alkylates guanines at both the C8-dG and *N*^2^-dG positions. The conformation of a site-specific *N*^2^-dG-IQ adduct in an oligodeoxynucleotide duplex containing the iterated CG repeat restriction site of the *Nar*I endonuclease has been determined. The IQ moiety intercalates, with the IQ H4a and CH_3_ protons facing the minor groove, and the IQ H7a, H8a and H9a protons facing the major groove. The adducted dG maintains the anti-conformation about the glycosyl bond. The complementary dC is extruded into the major groove. The duplex maintains its thermal stability, which is attributed to stacking between the IQ moiety and the 5′- and 3′-neighboring base pairs. This conformation is compared to that of the C8-dG-IQ adduct in the same sequence, which also formed a ‘base-displaced intercalated’ conformation. However, the C8-dG-IQ adopted the *syn* conformation placing the Watson−Crick edge of the modified dG into the major groove. In addition, the C8-dG-IQ adduct was oriented with the IQ CH_3_ group and H4a and H5a facing the major groove. These differences may lead to differential processing during DNA repair and replication.

## INTRODUCTION

Although browning meats during cooking imparts flavor, it also leads to the formation of heterocyclic amines (HCAs) such as 2-amino-3-methylimidazo[4,5-*f*]quinoline (IQ) (1–5). IQ has been produced in cooked meats at ppb levels (6,7) and has also been detected in tobacco smoke (8). HCAs and their metabolites have been isolated from human urine (9). Human exposures to HCAs, estimated to be ∼60 ng/day (10), are modest, but are likely to be involved in cancer etiology (11,12).

IQ is a potent mutagen (13). It is less prevalent than 2-amino-1-methyl-6-phenylimidazo[4,5-b]pyridine (PhIP) (14), but it is 200-fold more mutagenic in *Salmonella* reversion (Ames) assays (3) and it is an order of magnitude more mutagenic than aflatoxin B_1_. In these assays (15–18), HCAs such as IQ are active in point and frameshift tester strains (19). In bacteria, mutations occur primarily at G:C base pairs (20,21). IQ is a potent inducer of two-base frameshifts in CG repeats. Similar levels of mutations are seen in mammalian *hprt* (22) and *ef-2* (23) gene assays. Base-pair substitutions are the predominant mutations observed in mammalian cells (24–26). Sister chromatid exchange has been observed in rodent cells (27–29). IQ induces tumors in the organs of rodents and in the livers of monkeys (30–33). Liver, forestomach and lung tumors have been observed in IQ treated mice (34), whereas liver, intestine, zymbal gland, clitoral gland, skin (35), mammary glands, liver and ear ducts tumors have been observed in exposed rats (36). The TD_50_ values for IQ are 0.7 and 14.7 mg/kg/day in rats and mice, respectively (37). Human exposures to HCAs have been associated with pancreatic (38), colon (39), prostate (40) and breast cancers (41,42).

**Scheme 1.**
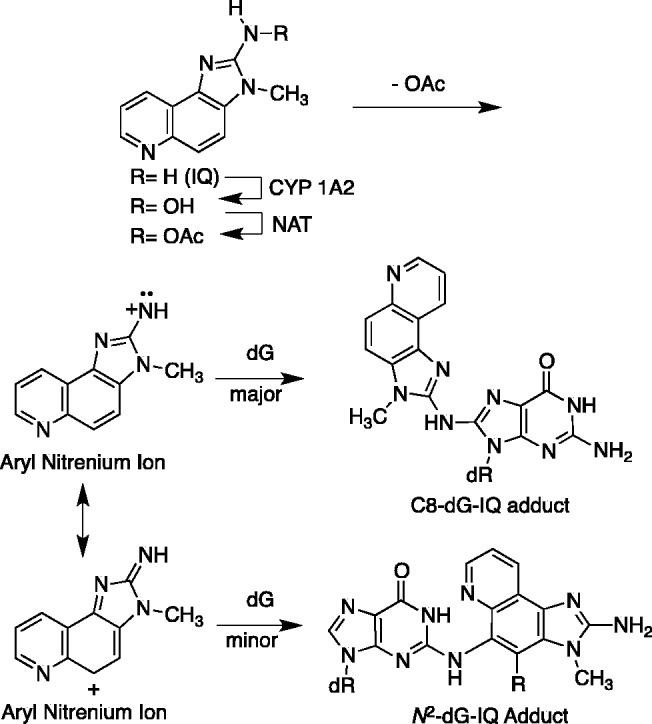
The bioactivation of IQ. Cytochrome P450-mediated *N*-hydroxylation followed by *N*-acetylation of the hydroxylamine and deacetylation forms an electrophilic aryl nitrenium ion. The aryl nitrenium ion alkylates guanine in DNA either via the IQ amine nitrogen to form the C8-dG-IQ adduct, or alternatively, alkylates DNA via the C5 position of the IQ ring to form the *N*^2^-dG-IQ adduct.

The genotoxicity of IQ derives primarily from its oxidation by CYP P450 1A2 to an *N*-hydroxylamine (43–47) although extra-hepatic CYP P450s oxidize HCAs with lower efficiencies (Scheme 1) (48). The *N*-hydroxylamine is acetylated by *N*-acetyl transferases, particularly NAT2 (49–51). In humans, the *NAT2* fast acetylator polymorphism correlates with increased genotoxicity and cancer (52–54). The nitrenium ion resulting from solvolysis of *N*-acetoxy-IQ is the ultimate electrophile (26,48). It reacts predominately at the C8 atom of guanine, while a minor alkylation product is formed at the *N*^2^ atom of guanine (55–57). In addition, IQ can be converted to a reactive and genotoxic *N*-nitrosamine, which shows similar regioselectivity for DNA alkylation (58,59).

Levels of C8 and *N*^2^-dG-IQ adducts have been measured in rat and primate tissues using mass spectrometry (60,61) and ^32^P post-labeling methodology. Turesky *et al.* (62) have monitored C8-dG-IQ adduct formation in human hepatocytes using tandem liquid chromatography-electrospray ionization mass spectrometry. Levels range from 7 to 26 adducts per 10^7^ bases. While less abundant, the *N*^2^-dG-IQ adduct is more persistent than is the C8-dG-IQ adduct, suggesting that it is repaired less efficiently (63). The *N*^2^-dG-IQ adduct may therefore play a significant role in the genotoxicity of IQ.

We have synthesized phosphoramidite reagents of the C8- and *N*^2^-dG-IQ adducts in which the Buchwald–Hartwig palladium-catalyzed *N*-arylation was the key C-N bonding-forming step (64–67). These adducts have been site-specifically incorporated into the *Nar*I restriction sequence, 5′-d(CG^1^G^2^CX^3^CC)-3′, using automated solid-phase synthesis. The G^3^ position is a hot spot for two-base frameshift deletions in bacterial mutagenesis assays, while the G^1^ position is not (68–71). In addition, human DNA polymerase (hpol) η produces two-base deletions when replicating past the *N*^2^-dG-IQ adduct at position G^3^, *in vitro* (72). Thus, this sequence provides a platform for investigating sequence-specific conformational perturbation of DNA structure by IQ adducts, in relationship to their biological processing (68–71,73,74). Previously, we determined the conformation of the C8-dG-IQ adduct at the G^3^ position of the *Nar*I sequence, which exhibited a base-displaced intercalated conformation (75).

**Chart 1.**
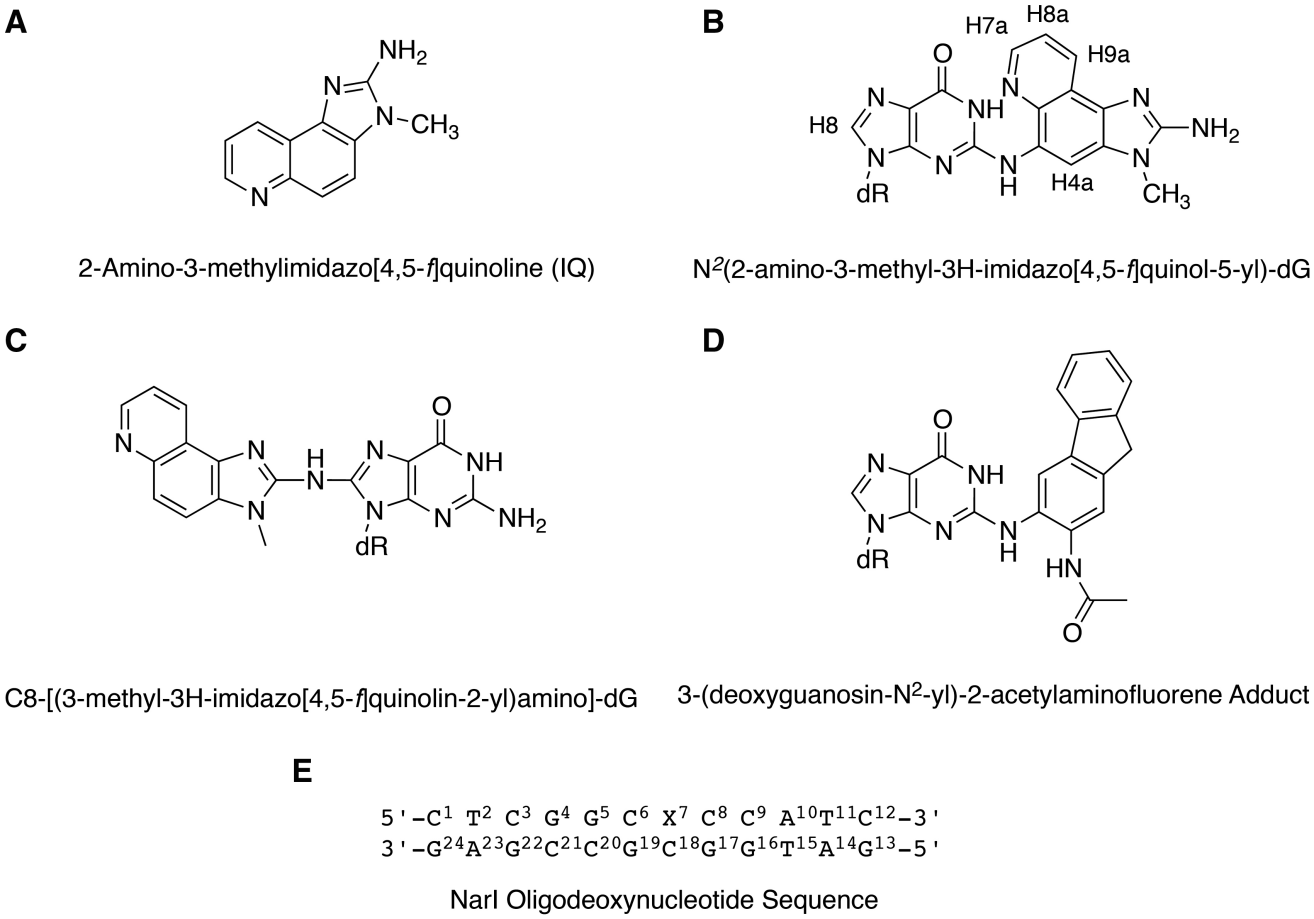
**(A)** Structure of IQ. **(B)** Structure of the *N*^2^-dG-IQ adduct, showing the numbering of guanine base and IQ protons. **(C)** Structure of the C8-dG-IQ adduct. **(D)** Structure of the *N*^2^-dG-AAF adduct. **(E)** The duplex containing the NarI sequence, showing the numbering of the nucleotides. The *N*^2^-dG-IQ adduct is positioned at X^7^, which corresponds to the G^3^ frameshift-prone position of the *Nar*I sequence.

Presently, we have determined the conformation of the *N*^2^-dG-IQ adduct in 5′-d(C^1^T^2^C^3^G^4^G^5^C^6^X^7^C^8^C^9^A^10^T^11^C^12^)-3′:5′-d(G^13^A^14^T^15^G^16^G^17^C^18^G^19^C^20^C^21^G^22^A^23^G^24^)-3′; X = *N*^2^-dG-IQ adduct (Chart 1). This duplex contains the recognition sequence of the *Nar*I restriction endonuclease, in which the G^3^ nucleotide (X^3^ in the *Nar*I sequence and X^7^ in this study) represents a hot spot for two-base deletions. The IQ moiety intercalates, with the IQ H4a and CH_3_ protons facing the minor groove, and the IQ H7a, H8a and H9a protons facing the major groove. The adducted nucleotide maintains the anti-conformation about the glycosyl bond. The complementary dC is extruded into the major groove. Nevertheless, the duplex maintains its thermal stability. This is attributed, in part, to stacking between the IQ moiety and the 5′- and 3′-neighboring base pairs. The base-displaced intercalated conformation of the *N*^2^-dG-IQ adduct differs from that of the C8-dG-IQ adduct, and may also be compared with that of the *N*^2^-dG AAF adduct (76), providing insight as to the persistence of the *N*^2^-dG-IQ adduct (63) and its processing during replication and repair events.

## MATERIALS AND METHODS

### Sample preparation

The *N*^2^-dG-IQ-adducted oligodeoxynucleotide 5′-d(CTCGGCXCCATC)-3′ was synthesized as described (67). The complement strand 5′-d(GATGGCGCCGAG)-3′ was synthesized by Midland Certified Reagents Co. (Midland, TX, USA) and purified by anion exchange chromatography. HPLC chromatographic utilized a Supelcosil LS-18-DB analytical base-deactivated C-18 column (Sigma-Aldrich Inc., St. Louis, MO, USA), using a gradient from 5% to 12% acetonitrile in ammonium formate buffer (pH 7), over 35 min, or a gradient from 5% to 30% acetonitrile in NaH_2_PO_4_ buffer (pH 5), over 25 min. The oligodeoxynucleotides were characterized by negative mode MALDI-TOF spectrometry in a hydroxypicolinic acid matrix. The oligodeoxynucleotides were annealed at 1:1 ratio at room temperature in 180 µl buffer containing 10 mM NaH_2_PO_4_, 100 mM NaCl and 5 µM Na_2_EDTA (pH 7).

### Thermal melting experiments

UV melting temperatures were collected on Cary 100 Bio UV spectrometer using 0.5 OD of duplex in 1 ml of solution containing 0.1 M NaCl, 10 mM NaH_2_PO_4_ and 0.05 mM Na_2_EDTA (pH 7.0). The temperature was increased from 25 to 75°C at a rate of 1°C per min.

### NMR spectroscopy

The *N*^2^-dG-IQ modified and the unmodified duplexes were prepared at concentrations of 570 and 810 µM, respectively, and placed into 3 mm diameter micro NMR sample tubes (77). The samples were prepared in 0.1 M NaCl, 50 µM Na_2_EDTA and 10 mM NaH_2_PO_4_ (pH 7.0). To observe non-exchangeable protons, the samples were exchanged with D_2_O. ^1^H NMR spectra were recorded at 600 or 800 MHz. The spectra were collected at 15°C; NOESY experiments were conducted at mixing times of 150, 200 and 250 ms with a relaxation delay of 1.8 s. Additional experiments were conducted with a longer relaxation delay to evaluate NOE distances arising from the adenine H2 protons, which typically exhibit longer *T*_1_ relaxation values. The data were collected with 512 points in the *t*1 dimension and 2048 points in the *t*2 dimension. Chemical shifts were referenced to water. For the observation of exchangeable protons, the samples were dissolved in 9:1 H_2_O:D_2_O. ^1^H NMR spectra were recorded at 600 or 800 MHz at 15°C. The data were collected with 512 points in the *t*1 dimension and 2048 points in the *t*2 dimension. A mixing time of 250 ms was used. Water suppression was performed using the WATERGATE pulse sequence (78). The spectra were processed using the TOPSPIN software (Bruker Biospin Inc., Billerica, MA, USA).

### NMR experimental restraints

The spectral data were evaluated using the program SPARKY (79). The intensities of NOE cross peaks were measured by volume integrations. The bounds for overlapped peaks were optimized manually. Noise was assigned half the intensity of the weakest peak, and motion was assumed to be isotropic. Experimental intensities were combined with intensities obtained from complete relaxation matrix analysis (CORMA) of starting model to generate a hybrid intensity matrix (80,81). The intensities were converted to distances with the program MARDIGRAS (82), which refined the hybrid intensity matrix. Calculations were performed using 150, 200 and 250 ms mixing time data and 2, 3 and 4 ns isotropic correlation times. Evaluation of the resulting distance data allowed creation of upper and lower bound distance restraints that were used in restrained molecular dynamics (rMD) calculations.

### Restrained molecular dynamics calculations

An unmodified B-DNA model (83) was used as a starting structure. The guanine at position G^7^ was replaced by the *N*^2^-dG-IQ adduct using the program INSIGHT II (Accelrys Inc., San Diego, CA, USA). Partial charges for the *N*^2^-dG-IQ adduct were calculated with the B3LYP/6-31 G* basis set in GAUSSIAN (84). The starting structure was energy minimized for 1000 cycles. Simulated annealing protocols (85) used for the rMD calculations were conducted with the parm99 force field (86), using the program AMBER (87). Force constants of 32 kcal/mol/Å^2^ were applied for distance and torsion angle restraints. The generalized Born model (88) was used for solvation. The salt concentration was 0.1 M. The molecule was coupled to the bath to control the temperature during simulated annealing (89). First, calculations were performed for 20 ps (20 000 steps) and recording data every ps by the following protocol: during steps 0–1000, the system was heated from 0 to 600 K with a coupling of 0.5 ps. During steps 1001–2000, the system was kept at 600 K. The system was then cooled from 600 to 100 K during steps 2001–18 000 with a coupling of 4 ps. Further cooling from 100 to 0 K occurred during steps 18 001–20 000 with a coupling of 1 ps. After initial cycles of refinement a longer 100 ps (100 000 steps) calculation was performed by the following protocol: During steps 0–5000, the system was heated from 0 to 600 K with a coupling of 0.5 ps. During steps 5001–10 000, the system was kept at 600 K. The system was cooled from 600 to 100 K during steps 10 001–90 000 with a coupling of 4 ps. Additional cooling from 100 to 0 K occurred during steps 90 001–100 000 with a coupling of 1 ps. Structure coordinates were saved after each cycle and were subjected to potential energy minimization. CORMA (80,81) was used to compare intensities calculated from these emergent structures with the distance restraints. Helicoidal analysis was performed using the CURVES+ web server (90,91).

## RESULTS

### Oligodeoxynucleotide containing the *N^2^*-dG-IQ adduct

The *N*^2^-dG-IQ adduct was incorporated into 5′-d(CTCGGCXCCATC)-3′ using automated solid-phase synthesis (67). The position of the *N*^2^-dG-IQ adduct was located at position X^7^, corresponding to position G^3^ in the *Nar*I sequence. The modified oligodeoxynucleotide was purified by C18 reverse phase HPLC and characterized by MALDI-TOF mass spectrometry in negative ion mode [*m/z* 3777.7, calcd for (M – H), 3776.6]. Thermal melting (*T*_m_) profiles of 0.5 *A*_260_ units of the IQ-modified duplex were monitored at 100 mM NaCl (1 ml volume) as a function of temperature by absorbance at 260 nm. An unmodified duplex was evaluated under the same conditions to provide a basis of comparison. The *T*_m_ of the modified duplex was 63°C, within experimental error of the unmodified duplex (Supplementary Figure S1). Thus, the *N*^2^-dG-IQ adduct did not reduce the stability of this oligodeoxynucleotide. This result differed from our previous report, which had indicated that this adduct destabilized this duplex (67). Subsequent analysis of the previous sample by mass spectrometry revealed that the complement strand was not correct, accounting for the discrepancy. Table 1 lists the correct *T*_m_ values of the *N*^2^-dG-IQ adduct at the three positions of the *Nar*I sequence.

**Table 1.** Thermal melting temperatures (*T*_m_ measurements) of NarI duplexes containing the *N*^2^-dG-IQ adducts

NarI *N*^2^-dG-IQ modified duplex	*T*_m_ (°C)	Δ*T*_m_* (°C)
5′-CTCXGCGCCATC-3′	62	−1
3′-GAGCCGCGGTAG-5′		
5′-CTCGXCGCCATC-3′	64	+1
3′-GAGCCGCGGTAG-5′		
5′-CTCGGCXCCATC-3′	63	0
3′-GAGCCGCGGTAG-5′		

### NMR

The modified duplex yielded well-resolved NMR spectra with narrow line shapes for the non-exchangeable protons at 15°C. The best spectral quality for the exchangeable protons was obtained at 5°C.

#### Non-exchangeable DNA protons

**Figure 1.**
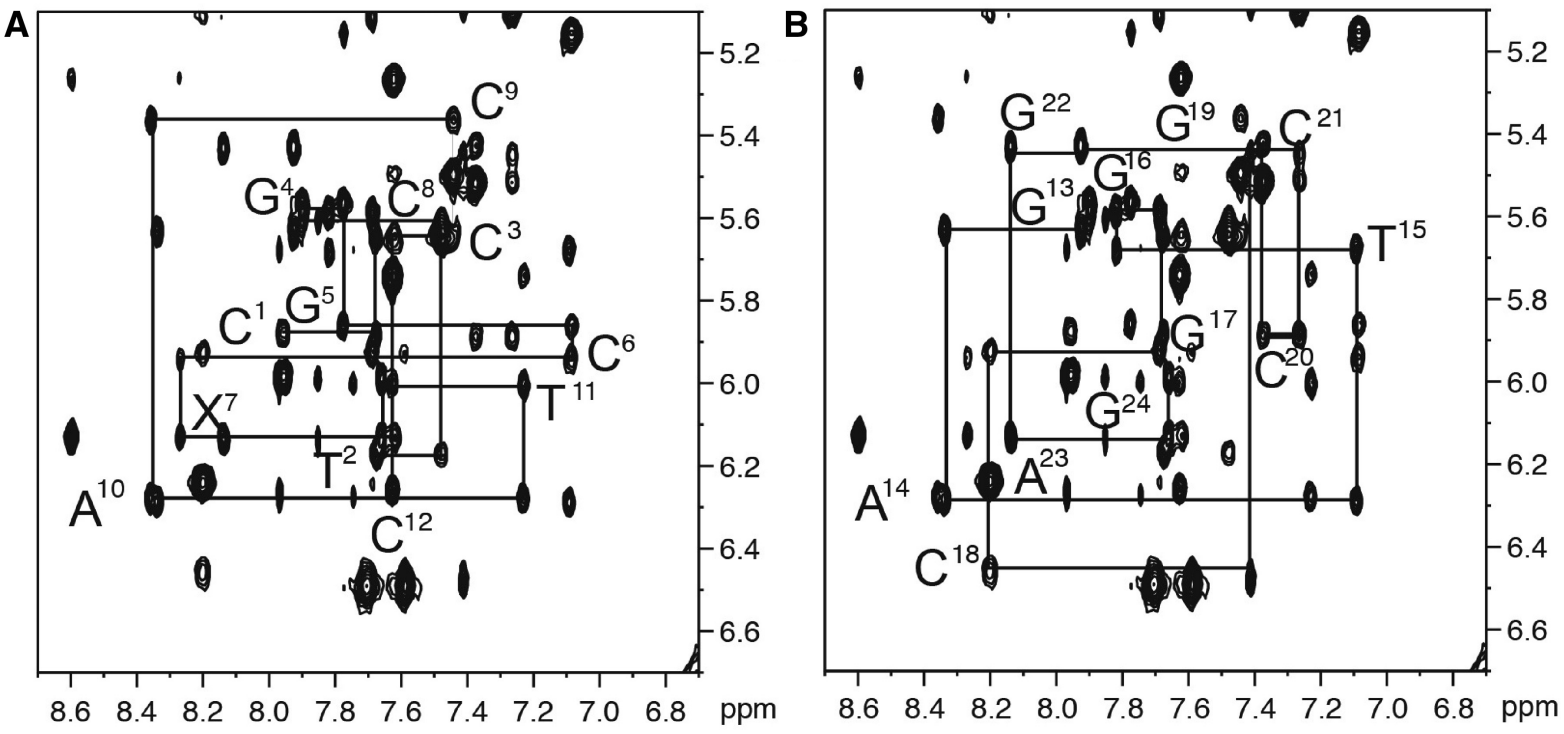
Expanded plot of the 250 ms NOESY spectrum showing NOEs between the base aromatic and deoxyribose anomeric protons of the *N*^2^-dG-IQ modified duplex. **(A)** Bases C^1^ to C^12^ of the modified strand. **(B)** Bases G^13^ to G^24^ of the complementary strand. The spectrum was acquired at 800 MHz at 15°C.

The base aromatic and deoxyribose anomeric protons were assigned using established procedures (Figure 1) (92,93). The intensity of the X^7^ H8 to X^7^ H1′ NOE was not changed in the presence of the adduct, indicating minimal change in the conformation of the glycosyl torsion angle. In the complementary strand, the intensity of the NOE between C^18^ H1′ and G^19^ H8 was weakened. The *N*^2^-dG-IQ adduct did not induce breaks in the sequential pattern of NOEs between the aromatic base protons and the anomeric protons. With the exception of the adduct site, the internucleotide NOEs were characteristic of a B-type duplex. The adenine H2 protons were assigned based upon NOEs to the thymine imino protons of the respective A:T base pairs. With the deoxyribose H1′ assignments in hand, the remainder of the deoxyribose protons was assigned from a combination of NOESY and COSY data. The assignments of the non-exchangeable DNA protons are summarized in Supplementary Table S1.

#### Exchangeable DNA protons

**Figure 2.**
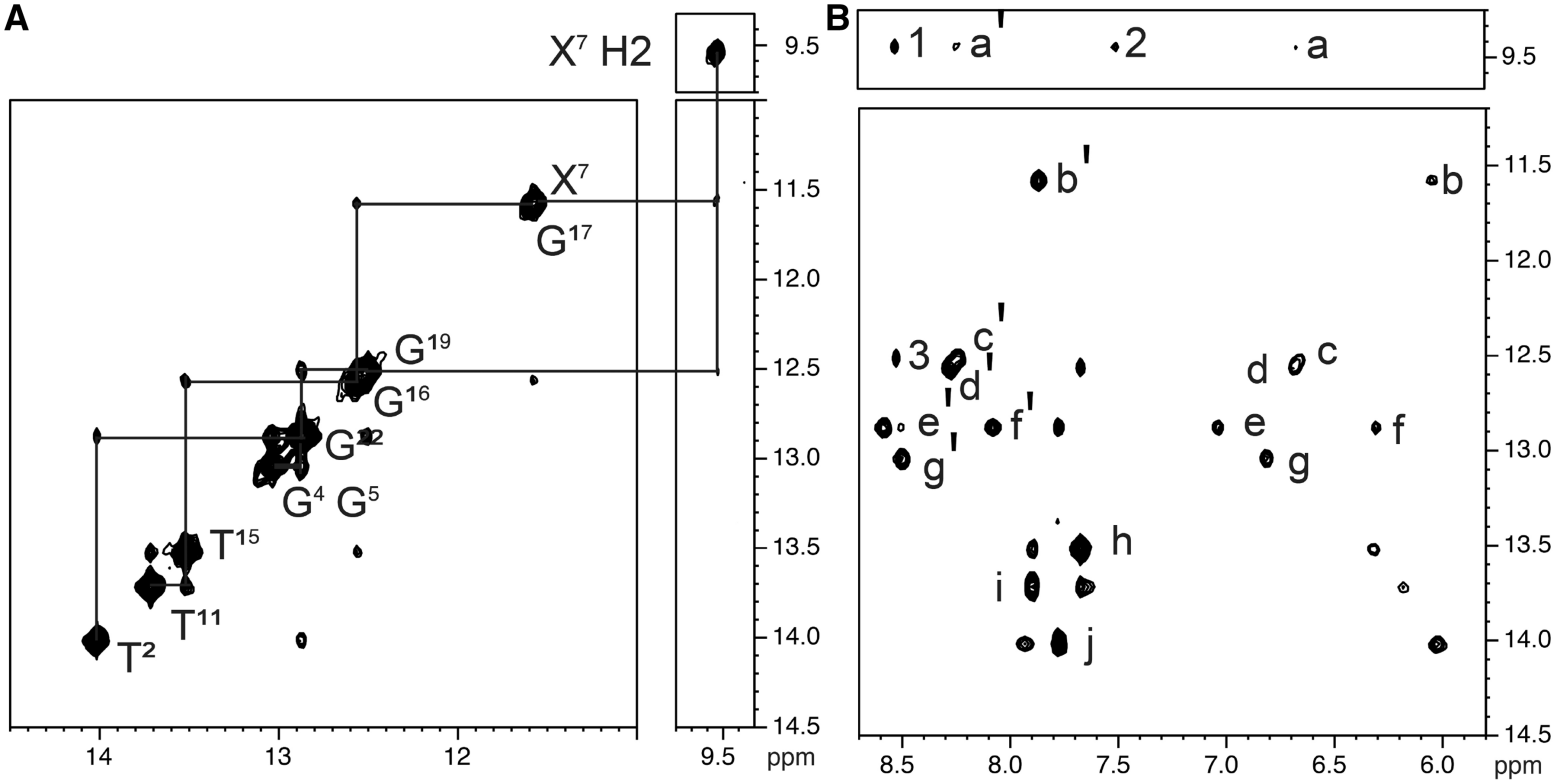
Expanded plots of the NOESY spectrum, showing the NOEs between the exchangeable imino and amino protons of the *N*^2^-dG-IQ-modified duplex. **(A)** Sequential NOE connectivity for the imino protons of base pairs T^2^:A^23^ to T^11^:A^14^. **(B)** NOE connectivity within Watson–Crick base pairs and between the imino protons and the amino protons. The lettered cross-peaks are assigned as follows: a′, X^7^ *N*^2^H →C^6^ *N*^4^Hb; a, X^7^ *N*^2^H →C^6^ *N*^4^Ha; b′, G^17^ N1H→C^8^ *N*^4^Ha; b, G^17^ N1H→C^8^ *N*^4^Hb; c′, G^19^ N1H→C^6^ *N*^4^Hb; c, G^19^ N1H→C^6^ *N*^4^Ha; d′, G^16^ N1H→C^9^ *N*^4^Hb; d, G^16^ N1H→C^9^ *N*^4^Ha; e′, G^22^ N1H→C^3^ *N*^4^Hb; e, G^22^ N1H→C^3^ *N*^4^Ha; f′, G^5^ N1H→C^20^ *N*^4^Hb; f, G^5^ N1H→C^20^ *N*^4^Ha; g′, G^4^ N1H→C^21^ *N*^4^Hb; g, G^4^ N1H→C^21^ *N*^4^Ha; h, T^15^ N3H→A^10^ H2; i, T^11^ N3H→A^14^ H2; j, T^2^ N3H→A^23^ H2; 1, X^7^ *N*^2^H→IQ H4a; 2, IQ H7a→X^7^ *N*^2^H; 3, X^19^ N1H→IQ H4a. The spectrum was collected at 800 MHz at 5°C.

The imino and amino proton regions of the NOESY spectrum are shown in Figure 2. The assignments were made using established methods (94). The *N*^2^-dG-IQ adduct perturbed Watson–Crick hydrogen bonding. At the X^7^:C^18^ base pair, the X^7^ imino proton resonance was broadened, probably due to an enhanced rate of exchange with water. The amino protons for C^18^ were not detected. No NOE was observed between the X^7^ and G^19^ imino protons, perhaps due to the broadening of the X^7^ imino proton. The chemical shifts of the X^7^ and G^17^ imino protons were almost isochronous. It was not possible to determine if a NOE between these two protons existed. All other base pairs were assigned, with the exception of the two terminal base pairs C^1^:G^24^ and C^12^:G^13^. The imino protons from the terminal base pairs were exchange broadened. Overall, the data suggested that the duplex maintained Watson–Crick hydrogen bonding, with the exception of the modified base pair (Figure 2B). The assignments of the exchangeable protons are summarized in Supplementary Table S2.

#### IQ protons

**Figure 3.**
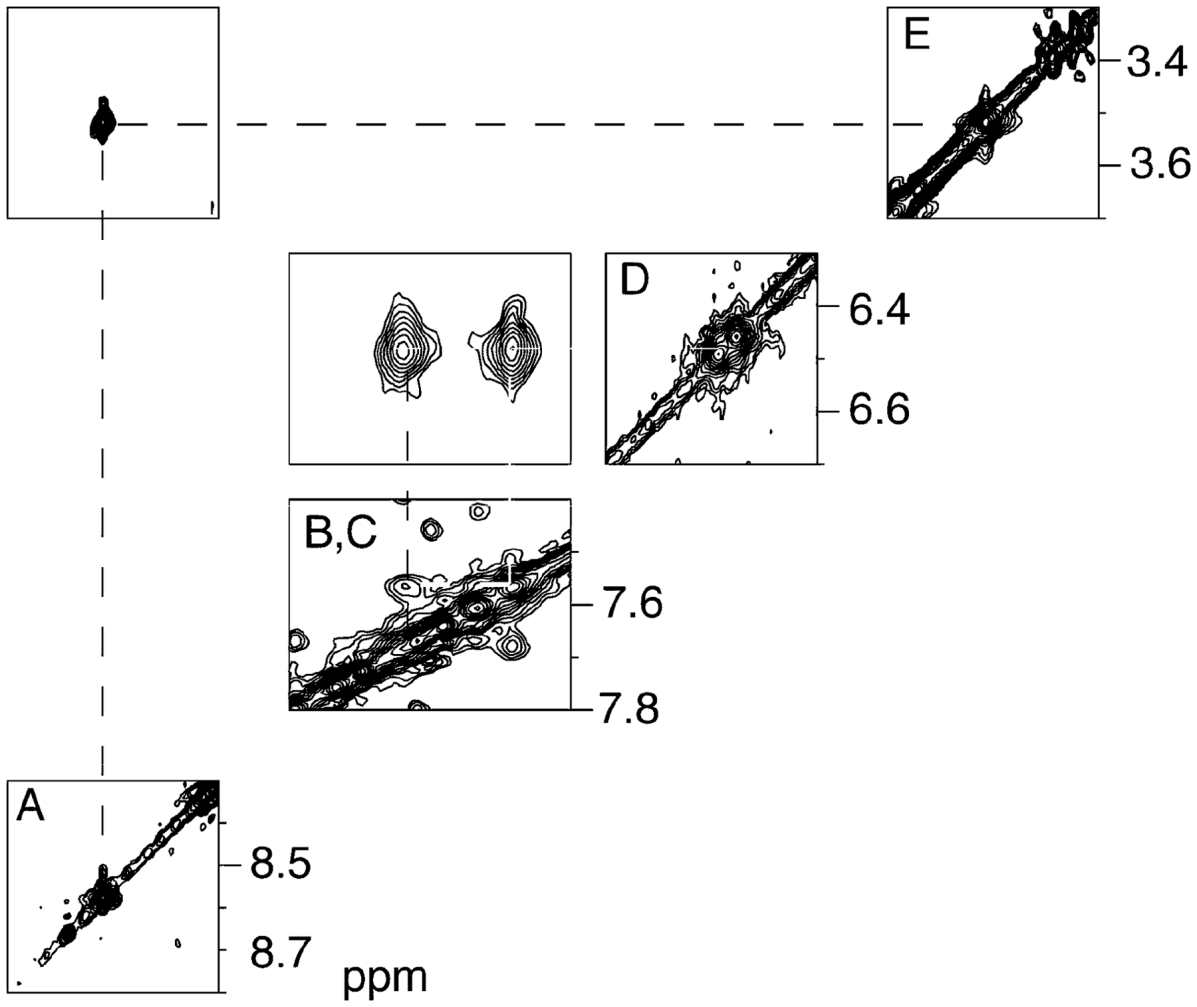
Expanded tile plot of the 250 ms NOESY spectrum showing the assignments of the IQ ring protons. **(A)** The IQ H4a proton was observed at 8.55 ppm. **(B)** The IQ H7a proton was observed at 7.65 ppm. **(C)** The IQ H9a proton was observed at 7.6 ppm. **(D)** The IQ H8a proton was observed at 6.55 ppm. **(E)** The IQ CH_3_ protons were observed at 3.57 ppm. The spectrum was collected at 800 MHz at 15**°**C.

The IQ protons, consisting of the CH_3_ group, the H4a proton, and the H7a, H8a and H9a spin system, were assigned from a combination of COSY and NOESY data (Figure 3). The CH_3_ resonance was observed at 3.57 ppm. It displayed an intense NOE to the H4a proton, whose resonance was observed at 8.55 ppm. A *^3^J *coupling between the H8a proton (δ 6.55 ppm) and the H9a proton (δ 7.65 ppm) was observed in the COSY spectrum. The H8a proton also exhibited an NOE to the H7a proton (δ 7.6 ppm). The ^3^*J *coupling between H8 and H7a exhibited weak intensity in the COSY spectrum. This was attributed to presence of the nitrogen atom in the ring, which broadened the H7a resonance. This effect was also observed for the C8-dG-IQ adduct, for which the COSY cross-peak between H7a and H8a was only observed between 25 and 35°C (95). The IQ amine proton was not assigned.

#### Chemical shift perturbations

**Figure 4.**
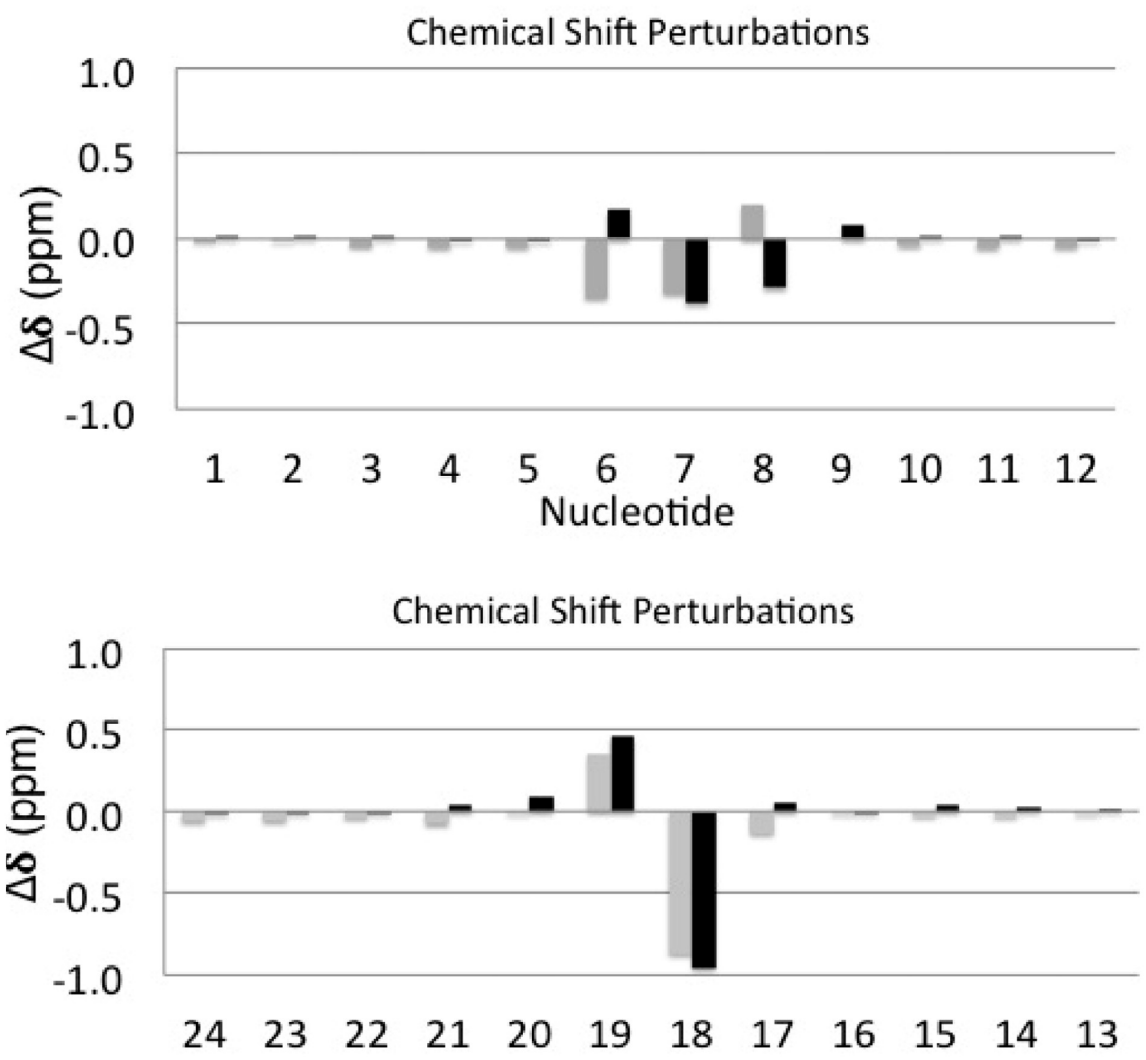
Chemical shift perturbations of the deoxyribose H1′ protons (gray) and the pyrimidine H6 or purine H8 aromatic protons (black), for the *N*^2^-dG-IQ modified duplex. **(A)** Nucleotides C^1^–C^12^ in the modified strand. **(B)** Nucleotides G^13^–G^24^ in the complementary strand. The Δδ (ppm) values were calculated as δ_modified duplex_–δ_unmodified_ duplex. Positive Δδvalues represent upfield chemical shift perturbations. Negative Δδvalues represent downfield chemical shift perturbations.

The *N*^2^-dG-IQ adduct resulted in localized chemical shift perturbations, involving the modified base pair X^7^:C^18^ and the neighboring C^6^:G^19^ and C^8^:G^17^ base pairs (Figure 4). At the modified X^7^:C^18^ base pair, the X^7^ H8 resonance shifted 0.4 ppm downfield relative to the G^7^ H8 resonance in the unmodified duplex. In contrast, the C^18^ H6 and C^18^ H1′ resonances shifted 1 and 0.8 ppm downfield, respectively. At the 5′-neighbor C^6^:G^19^ base pair, the C^6^ H6 resonance shifted upfield by 0.2 ppm, whereas the C^6^ H1′ resonance shifted downfield by 0.4 ppm. The G^19^ H8 and H1′ resonances each shifted upfield by 0.4 ppm. At the 3′-neighbor C^8^:G^17^ base pair, the C^8^ H6 resonance shifted downfield by 0.3 ppm, whereas the C^8 ^H1′ resonance shifted upfield by 0.2 ppm. The G^17^ H8 resonance shifted downfield by 0.1 ppm. The G^17^ H1′ resonance showed negligible chemical shift perturbation. The resonances for the remaining base pairs in the duplex also showed negligible chemical shift perturbations. In the imino proton region of the spectrum, the X^7^ and G^17^ N1H imino resonances, at 11.57 and 11.59 ppm, respectively, exhibited upfield chemical shifts of >1 ppm from those of the unmodified duplex, at 13.24 and 13.16 ppm, respectively. The X^7^ *N*^2^H amine resonance was observed at 9.5 ppm.

#### NOEs between IQ and DNA

The CH_3_, H4a, H7a and H8a protons of IQ exhibited NOEs to the C^8^, G^17^, C^18^ and G^19^ bases (Table 2). The pattern of NOEs involving H9a was difficult to establish due to resonance overlap with G^17^. The CH_3_ group showed medium strength NOEs to X^7^ H1′ and C^8^ H6 and weak NOEs to C^8^ H1′ and C^8^ H5. It also showed an NOE to the G^19^ N1H imino proton. The H4a proton showed a strong NOE to X^7^ H1′, and medium NOEs to C^8^ H5, the X^7^ N2H amine proton and the G^19^ N1H imino proton and weak NOEs to X^7^ H2′ and X^7^ H2′′ The H7a proton exhibited weak NOEs to G^17^ H1′, and the X^7^ N2H amine proton. Three NOEs from X^7 ^to C^18^ were observed; these were of medium strength NOE between H9a and C^18^ H1′, and H8a and medium strength between C^18^ H2′ and H2′′. The H8a proton showed a medium strength NOE to G^19^ H3′ and weak NOEs to G^17^ H1′ and G^19^ H8. Some 30% of the NOEs from the IQ ring were to protons in the complementary strand, whereas another 46% were to other IQ protons and protons of the modified base. The remaining 24% of the NOEs were to neighbor bases in the modified strand.

**Table 2.** Summary of NOEs observed between *N*^2^-dG-IQ(X7) adduct protons and oligodeoxynucleotide protons and their intensities

IQ proton	NOEs to oligodeoxynucleotide protons
CH_3_	X^7^ H1′: medium; C^8^ H6: medium; C^8^ H1′: weak; C^8^ H5: weak
H4a	X^7^ H1′: strong; C^8^ H5: medium; X^7^ H2′: weak; X^7^ H2′′: weak; X^7^ N2H: medium; G^19^ N1H: medium
H7a	G^17^ H1′: weak; X^7^ H2: weak
H8a	G^19^ H3′: medium; C^18^ H2′: medium; C^18^ H2′′: medium; G^17^ H1′: weak; G^19^ H8: weak; G^17^ H2′′: weak
H9a	C^18^ H1′: medium

### Conformational Refinement

After the unmodified duplex was constructed using B-DNA coordinates (83), the guanine at position G^7^ was replaced by the *N*^2^-dG-IQ adduct. The partial charges for the *N*^2^-dG-IQ adduct are provided in Supplementary Figure S2. Potential energy minimization provided an energy minimized starting duplex. A total of 329 distance restraints consisting of 127 inter- and 202 intra-nucleotide distances (Table 3) were obtained using the program MARDIGRAS (81,82), from 15°C NOESY data. Similar distance restraints were obtained if the data were collected at 150, 200 or 250 ms mixing times. These restraints included 16 DNA-IQ distances. A total of 49 Watson–Crick hydrogen-bonding restraints were applied for all of the base pairs except for the modified X^7^:C^18^ base pair. An additional 100 phosphodiester backbone and 20 deoxyribose pseudorotation restraints for base pairs not proximal to the site of modification were obtained from canonical values derived from B-DNA (83), consistent with the spectroscopic data indicating that the duplex maintained a B-DNA like structure. A series of rMD calculations were performed using a simulated annealing protocol in which the generalized Born solvation model (88) was used, with a salt concentration of 0.1 M. The emergent structures were subjected to potential energy minimization before further analysis, which involved a 100 ps rMD calculation using the protocol described above, again followed by potential energy minimization.

**Table 3.** NMR restraints used for the *N*^2^-dG-IQ structure calculations and refinement statistics

NOE restraints	
Internucleotide	127
Intranucleotide	202
Total	329
Backbone torsion angle restraints	100
H-bonding restraints	49
Deoxyribose restraints	20
Total number of restraints	498
Refinement statistics	
Number of distance restraint violations	56
Number of torsion restraint violations	50
Total distance penalty/maximum penalty (kcal/mol)	2.3/0.187
Total torsion penalty/maximum penalty (kcal/mol)	2.8/0.177
r.m.s. distances (Å)	0.012
r.m.s. angles (°)	2.5
Distance restraint force field (kcal/mol/Å^2^)	32
Torsion restraint force field (kcal/mol/deg^2^)	32

**Figure 5.**
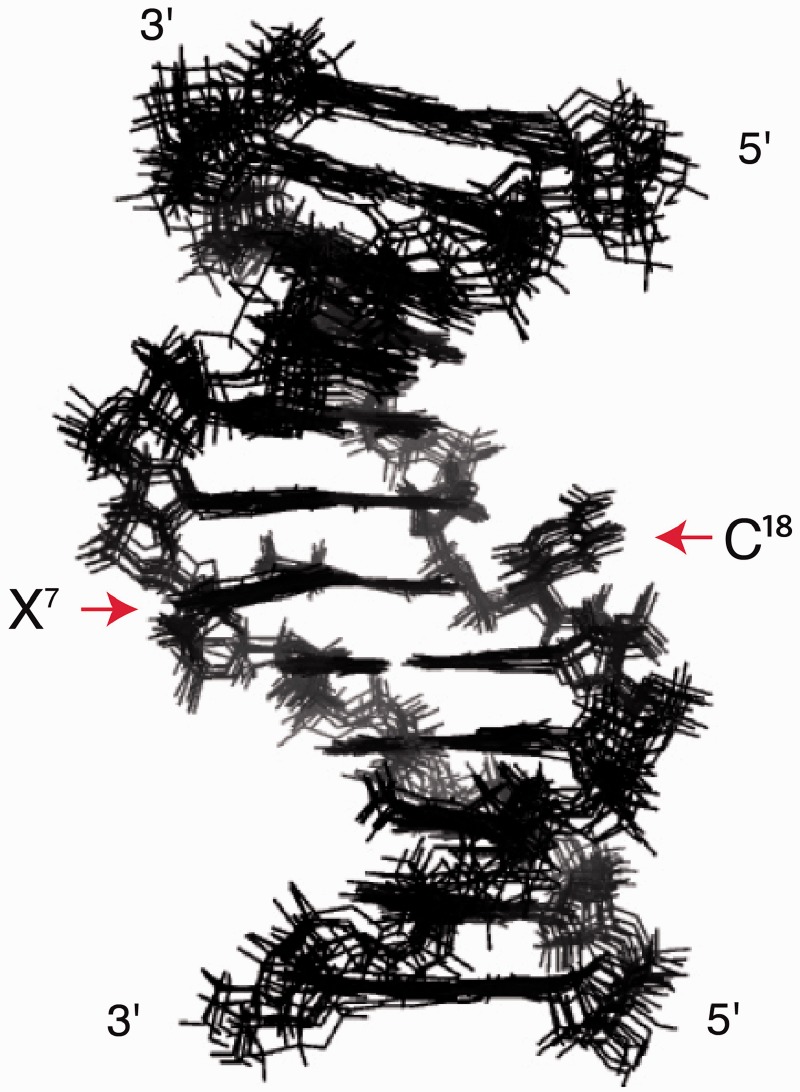
Superposition of ten potential energy minimized structures emergent from the rMD calculations of the *N*^2^-dG-IQ modified duplex, using distance restraints from the 250 ms NOESY data. The positions of the modified X^7^ nucleotide and the C^18^ nucleotide in the complementary strand are as indicated. The maximum pairwise rmsd between these 10 structures was 1.12 Å.

The pairwise rmsd analysis of structures emergent from the rMD calculations was used to measure the precision of the structural refinement. Ten structures were chosen based on the lowest deviations from the experimental distance and dihedral restraints (Figure 5). These exhibited an rmsd of 0.012 Å in distances and 2.5° in torsion angles (Table 3). There were 56 distance violations with a maximum penalty of 0.187 kcal/mol and a total distance penalty of 2.3 kcal/mol. There were 50 torsion angle violations with a maximum penalty of 0.177 kcal/mol and a total torsion angle penalty of 2.8 kcal/mol. The maximum pairwise rmsd distances were 1.12 Å. These structures were averaged and subjected to potential energy minimization.

**Figure 6.**
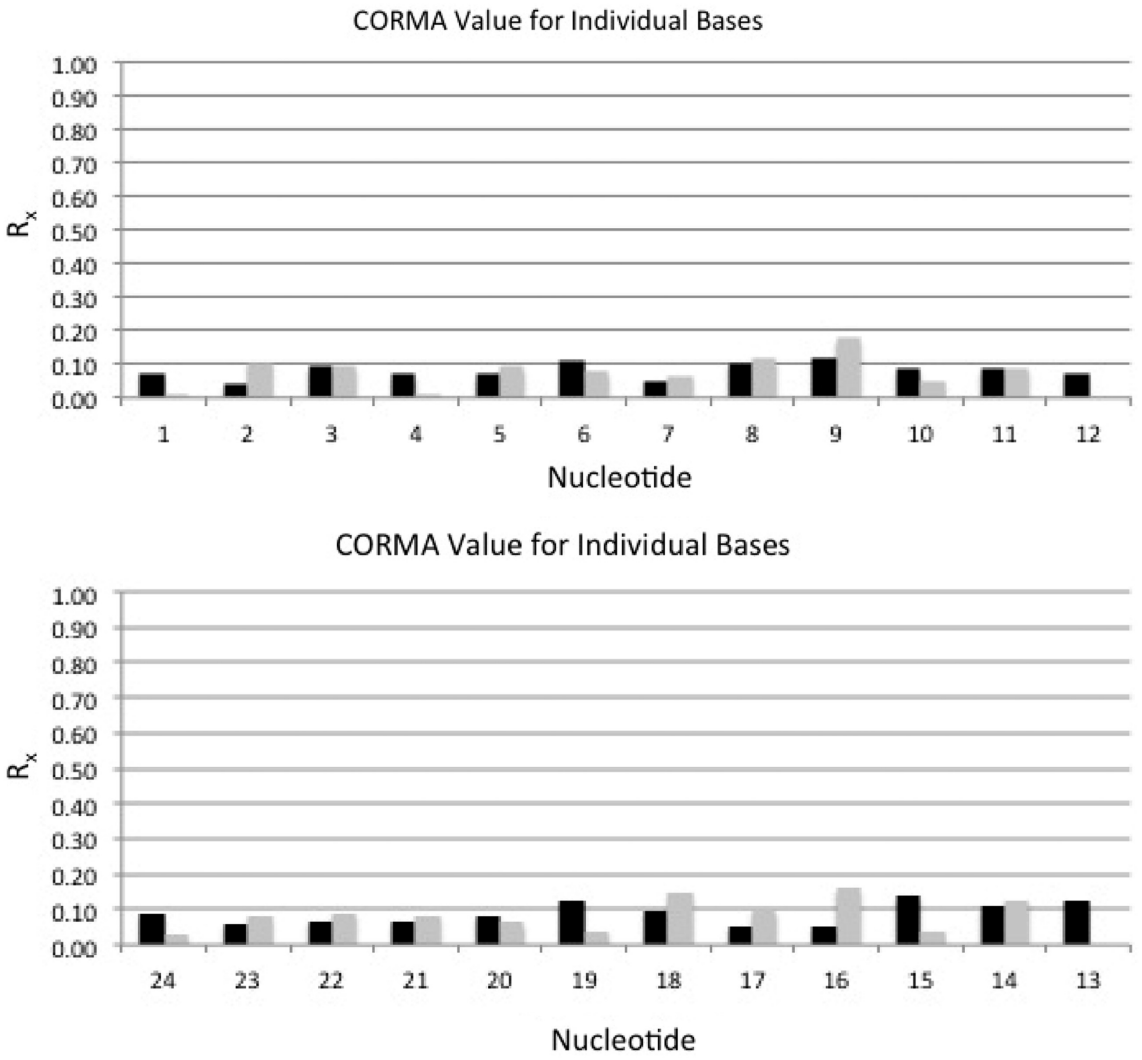
Sixth root residuals (*R*^1^_x_) calculated using complete relaxation matrix calculations from the average of 10 structures emergent from the rMD calculations of the *N*^2^-dG-IQ modified duplex. The black bars represent intra-nucleotide sixth root residuals, and the gray bars represent inter-nucleotide sixth root residuals. **(A)** Nucleotides C^1^–C^12^ in the modified strand. **(B)** Nucleotides G^13^–G^24^ in the complementary strand.

The accuracy of the refined structures was assessed by complete relaxation matrix analyses (80,81), which compared intensities calculated from the refined structures with the distance restraints (Figure 6). The sixth root residual *R*^1^_x_ value of the average structure was 8.4%, and the individual values for intra-nucleotide restraints (8.5%) and inter-nucleotide restraints (8.3%) were of similar magnitudes. This indicated agreement with the NOE data. Nucleotide G^19^ exhibited a greater *R*^1^_x_ value of 17.1%, suggesting that it was not as well-refined. This was attributed to several NOEs involving G^19^ being overlapped with other resonances. The structural statistics are summarized in Table 4.

**Table 4.** Structural statistics for the *N*^2^-dG-IQ modified duplex

Average structure (obtained from 10 structures)			
RMS pairwise difference between structures		1.12	
RMS difference from average structure		0.75	

	CORMA analysis for average structure^a^		
	Intranucleotide	Internucleotide	Total

*R*^1^_x_^b^	0.085	0.083	0.084
Average error^c^			0.0037

^a^The mixing time was 250 ms.

^b^R^1^_x_ is the 6^th^ root R factor: 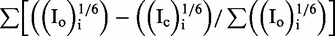.

^c^Average error: 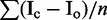, where the I_c_ are NOE intensities calculated from the refined structure, the I_o_ are experimental NOE intensities.

### Conformation of the *N*^2^-dG-IQ Adduct

**Figure 7.**
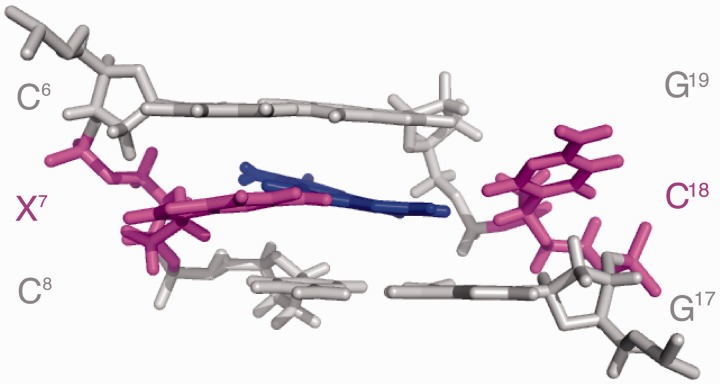
Expanded view of the average structure calculated from 10 structures emergent from the rMD calculations of the *N*^2^-dG-IQ(X^7^) modified duplex, showing base pairs C^6^:G^19^, X^7^:C^18^ and C^8^:G^17^. The view is from the major groove. The modified base pair X^7^:C^18^ is shown in magenta, with the IQ moiety shown in blue.

**Figure 8.**
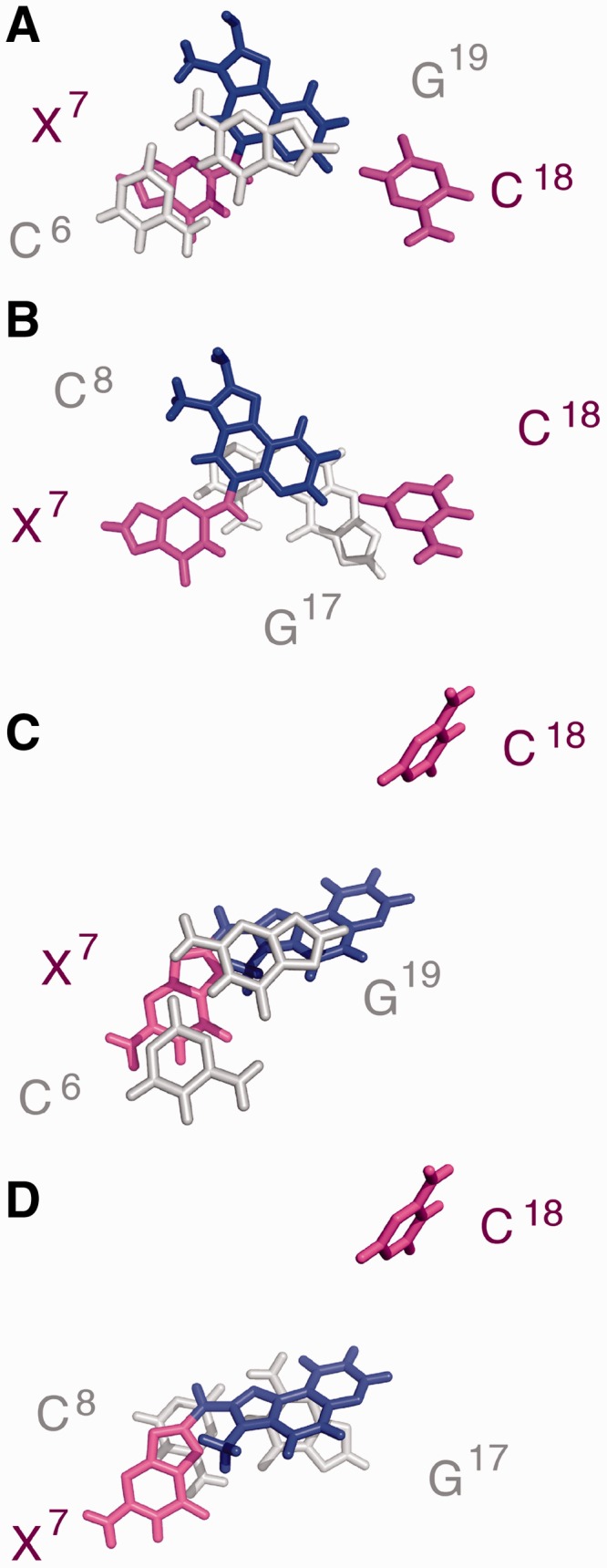
Expanded views of the average structure calculated from 10 structures emergent from the rMD calculations of the *N*^2^-dG-IQ(X^7^) modified duplex. Base stacking of the modified X^7^:C^18^ base pair with the 5′-neighbor and 3′-neighbor base pairs. **(A)** Stacking of C^6^:G^19^ above X^7^:C^18^. **(B)** Stacking of X^7^:C^18^ above C^8^:G^17^. Expanded views of the average structure of the corresponding C8-dG-IQ adduct at X^7^. (75) **(C)** Stacking of C^6^:G^19^ above X^7^:C^18^. **(D)** Stacking of X^7^:C^18^ above C^8^:G^17^. In each instance, the modified base pair X^7^:C^18^ is shown in magenta, with the IQ moiety shown in blue.

The modified nucleotide (X^7^) remained in the anti-conformation about the glycosyl bond. It was displaced toward the major groove. The IQ ring was intercalated and oriented such that the H4a proton and the CH_3_ group faced into the minor groove, whereas the H7a, H8a and H9a protons faced into the major groove (Figure 7). The IQ ring was angled by ∼15° with respect to the modified guanine, but otherwise remained largely in plane with the damaged base. The helix was unwound between C^6^ and X^7^, with a reduced helicoidal twist of 30°. This was partially compensated by an increased twist of 9° between X^7^ and C^8^. At base pair X^7^:C^18^, the roll of the X^7^ purine decreased by 24°. This was compensated at base pair C^8^:G^17^, where the roll decreased by 12°. Consequently, the *N*^2^-dG-IQ adduct induced a bend of 10° to the duplex. The IQ ring exhibited stacking with the flanking base pairs (Figure 8). IQ was stacked between G^17^ and G^19^ of the complementary strand of the C^6^:G^19^ and C^8^:G^17^ base pairs. The complementary nucleotide, C^18^, extruded into the major groove and did not exhibit stacking with the neighboring base pairs. The base opening between X^7^ and C^18^ increased by 76°. This disrupted Watson–Crick hydrogen bonding. The other base pairs maintained Watson–Crick hydrogen bonding.

## DISCUSSION

The *N*^2^-dG-IQ DNA adduct has been of interest following reports that it is more persistent than the C8-dG-IQ adduct in rodents and primates that were fed IQ in their diet (63). The synthesis of this adduct into oligodeoxynucleotides (67) has allowed the conformation of the *N*^2^-dG-IQ adduct at the G^3^ position of this sequence to be determined. This is a hot spot for two-base frameshift deletions in bacterial mutagenesis assays (68–71,73,74). In addition, human DNA polymerase (hpol) η produces two-base deletions when replicating past the *N*^2^-dG-IQ adduct at the reiterated G^3^ position of the NarI sequence, *in vitro* (72).

### Conformation of the *N*^2^-dG-IQ adduct

The IQ ring intercalates when the *N*^2^-dG-IQ adduct is positioned at the frameshift-prone G^3^ position of the NarI sequence (Figure 7). The strong NOE intensities of the IQ H4a and CH_3_ protons to the X^7^ and C^8^ H1′ protons (Table 1) indicate that these protons face into the minor groove and establish the conformation about the bond between *N*^2^-dG and C5 of the IQ moiety. In contrast, NOEs involving the H8a proton of the IQ ring are primarily to bases G^17^, C^18^ and G^19^ of the complementary strand (Table 1). The chemical shifts of the IQ H7a, H8a and H9a protons are observed between 6.5 and 8.0 ppm, which is 1.3–2.0 ppm upfield as compared to the *N*^2^-IQ-dG nucleoside. This is consistent with the intercalated conformation and stacking of the IQ ring below the 5′-neighboring G^19^ of the complementary strand and above the 3′-neighboring C^8^:G^17^ base pair (Figure 8). Chemical shift perturbations corroborate the NOE data (Figure 4). The IQ H4a proton resonance, observed at 9.6 ppm, is 0.4 ppm upfield from the resonance observed for the modified *N*^2^-dG-IQ nucleoside (67), consistent with its location below G^19^ and above C^8^ (Figure 8). The IQ moiety displaces the complementary C^18^ base from the duplex, and flips it into the major groove. This is supported by smaller perturbations in chemical shifts for the H4a and CH_3_ protons as compared to the H7a, H8a and H9a aromatic protons of IQ. The displacement of the modified nucleotide X^7^ toward the major groove (Figure 8) is supported by the downfield chemical shift change of 0.4 ppm for the X^7^ H8 and H1′ protons of the modified base. The C^8^ H6 proton resonance also experiences a downfield shift of 0.3 ppm. The stacking interactions of the IQ ring with the flanking bases C^8^, G^17^ and G^19^ are reflected in the thermodynamic analysis of the adduct, in which the thermal melting temperature of 63°C is unchanged from that of the unmodified duplex.

### Comparison to the *N*^2^-acetylaminofluorene-dG adduct

The other *N*^2^-dG arylamine adduct that has been subjected to conformational analysis, although not in the *Nar*I sequence of interest herein, is that arising from *N*-acetylaminofluorene (AAF; Chart 1) (76). The *N*^2^-dG-AAF adduct conformation has also been examined using computational approaches (96). Zaliznyak *et al.* (76) have shown that the AAF moiety resides in the minor grove with its long axis directed toward the 5′-end of the modified strand. This shields the hydrophobic AAF ring from water. Similar to the *N*^2^-dG-IQ adduct, the modified nucleotide maintains the anti-conformation about the glycosyl bond. Notably, the *N*^2^-dG-AAF adduct increases the stability of the DNA, which has been attributed to a favorable entropic effect (76). The present data reveal that the base-displaced intercalated conformation of the *N*^2^-dG-IQ adduct at position G^3^ of the *Nar*I sequence differs from that of the *N*^2^-dG-AAF adduct, suggesting that the conformations of *N*^2^-dG arylamine adducts vary rather than following a common motif. At the molecular level, the factors governing whether planar aromatic molecules such as AAF or IQ favor DNA groove binding versus intercalation are not well established, but may be influenced both by their electronic structures and their respective geometries (97). Replication bypass studies have revealed that the *N*^2^-dG-AAF adduct largely blocked DNA synthesis, but with some bypass and mis-incorporation of dATP opposite the lesion (98).

### Comparison to the C8-dG-IQ adduct

When the C8-dG-IQ adduct was placed into the *Nar*I sequence at the frameshift-prone G^3^ position, the IQ ring also intercalated into the duplex and the complementary C^18^ base was extruded into the major groove. The conformation of the C8-dG-IQ adduct also was characterized as base-displaced intercalated (75). Thus, at the G^3^ position within the *Nar*I sequence, both the C8-dG-IQ and *N*^2^-dG-IQ adducts share a motif in which the IQ ring intercalates and C^18^ is extruded into the major groove. However, the two conformations are distinctive. Apart from the difference in the regiochemistry of alkylation (C8 versus *N*^2^; Scheme 1), a major difference between the C8-dG-IQ and *N*^2^-dG-IQ adducts is that the C8-dG-IQ-modified guanine adopts a *syn* conformation about the glycosyl bond, whereas the *N*^2^-dG-IQ-modified guanine maintains the anti-conformation about the glycosyl bond (Figures 8 and 9). In addition, for the C8-dG-IQ adduct, rotation of the glycosyl bond into the *syn* conformation places the Watson−Crick hydrogen bonding edge of the modified dG into the major groove. The X^7^ imino and amino protons are exposed to solvent. For the C8-dG-IQ adduct the orientation of the IQ ring with respect to the base is opposite to that of the *N*^2^-IQ adduct, such that the IQ CH_3_ group and H4a and H5a protons face the major groove rather than the minor groove (75). The orientation of the C8-dG-IQ adduct in the duplex rotates the bulk of the IQ aromatic ring away from the flanking bases, resulting in a loss of base-stacking interactions, as shown in Figure 8. In comparison, the *N^2^*-dG-IQ adduct appears to have more favorable stacking interactions with G^19^. These differences may lead to differential processing during both DNA repair and DNA replication.

### Structure–activity relationships

The *N*^2^-dG-IQ adduct is less efficiently removed from genomic DNA by nucleotide excision repair (63,99). The NER machinery is thought to recognize bulky DNA damage that is destabilizing and distortive to the duplex (100–103). It has been proposed that the thermal stabilization of the *N*^2^-dG-AAF adduct hinders NER (76). We observe that the *T*_m_ of the *N*^2^-dG-IQ adduct at position G^3^ within the *Nar*I sequence does not destabilize the duplex (Table 1), correcting our original report (67). The *T*_m_ of the *N*^2^-dG-IQ modified duplex is 63°C, and does not differ significantly from the unmodified duplex. This is remarkable given that the intercalated IQ moiety disrupts Watson–Crick hydrogen bonding and that the complementary C^18^ base is displaced into the major groove. The stability of the *N*^2^-dG-IQ modified duplex likely arises from favorable stacking between the IQ moiety and the neighboring base pairs (Figure 8). It is also interesting to note that unlike the *N^2^*-dG-IQ adduct, the C8-dG-IQ adduct, which does not stack with the neighboring bases as well at this position (Figure 8) thermally destabilizes the duplex, reducing the *T*_m_ by 4°C. Yeo *et al.* (104) examined AAF and AF C8-dG adducts within the *Nar*I sequence and observed a correlation between the degree of destabilization induced by the lesions, binding affinities to the damage recognition protein XPC-RAD23B and overall NER efficiencies. Likewise, Zaliznyak *et al.* (76) attributed the increased stability of the *N*^2^-dG-AAF adduct to its orientation within the minor groove and the entropy-favored release of waters from the duplex. Similar conclusions were reached by Cai *et al.* (105) who correlated thermodynamic stabilities and van der Waals interaction energies with repair efficiencies for stereoisomeric intercalated *N*^6^-dA PAH adducts. Their studies showed that intercalated adducts with fewer DNA structural distortions and increased van der Waals interactions with neighboring bases correlated with reduced repair efficiencies. The HCA PhIP adduct has been compared with the *cis*-B[*a*]P-*N*^2^-dG adduct in duplex DNA and in a nucleotide deletion duplex, and it was concluded that local stabilization induced by these adducts governs the ability of the β-hairpins of NER proteins to recognize the damage (106). In summary, it seems plausible that the thermal stability of the *N*^2^-dG-IQ adduct may, in part, explain the persistence of the *N*^2^-dG-IQ adduct in rats and primates.

If not repaired, the *N*^2^-dG-IQ adduct is anticipated to be genotoxic. Indeed, IQ is an order of magnitude more mutagenic than is aflatoxin B_1_ in Ames assays. The mutations occur primarily at G:C base pairs (20,21). The replication of the *N*^2^-dG-IQ-adduct within the *Nar*I sequence is influenced by the identity of the DNA polymerase. Because the damaged guanine remains in the anti-conformation about the glycosyl bond (Figures 7 and 8), one might anticipate that the *N*^2^-dG-IQ lesion should block Watson–Crick base pairing with incoming dNTPs during lesion bypass. Stover *et al.* (107) incorporated the *N*^2^-dG-IQ-adduct into the G^1^ and G^3^ positions of the *Nar*I sequence and examined replication of the oligodeoxynucleotides with *Escherichia coli* polymerases (pol) I (exonuclease deficient Klenow fragment), exonuclease deficient pol II and the *Solfolobus solfataricus* P2 DNA polymerase IV (Dpo4), *in vitro*. At the G^3^ position, the *N*^2^-dG-IQ adduct blocked the *E. coli* polymerases. Pol II *exo*^−^ favored correct incorporation of dCTP over dGTP but was unable to extend either of these initial insertion products. In contrast, the Dpo4 polymerase bypassed the *N*^2^-dG-IQ adduct and produced an error-free product. The present studies do not necessarily predict the structure of the *N*^2^-dG-IQ adduct during trans-lesion bypass. Consequently, it will be of interest to prepare complexes of bypass polymerases with *N*^2^-dG-IQ modified template:primers in an effort to determine how the *N*^2^-dG-IQ adduct is accommodated during lesion bypass and how polymerases, e.g. the Dpo4 polymerase (108) allow bypass of this lesion.

Bypass of the *N*^2^-dG-IQ adduct has been reported to be dependent upon its position in the *Nar*I sequence. Choi *et al.* (72) have demonstrated that the human DNA polymerase (hpol) η can extend primers beyond template *N*^2^-dG-IQ adducts. Pol η correctly inserts dCTP and incorrectly inserts dATP. Analyses of hpol η extension products reveal that a −2 bp deletion occurs with the G^3^ *N*^2^-dG-IQ adduct. In contrast, at the G^1^ position replication past the *N*^2^-dG-IQ adduct results in error-free incorporation of dCTP, but further extension is inhibited and the polymerase stalls. In contrast, hpol η does not yield −2 bp deletions with the C8-dG-IQ adduct located at position G^3^. While further studies will be necessary to probe the basis for these observations, it is of interest to note that the stability of the *N*^2^-dG-IQ adduct placed opposite a 2-bp deletion increases as compared to the fully complementary duplex, suggesting that the adduct may stabilize a 2-bp strand slippage intermediate (67). At the G^1^ position, the *N*^2^-dG-IQ adduct is bypassed and extended by the *E. coli* polymerases and the Dpo4 polymerase, and error-free product is observed. Thus, it will also be of interest to examine the structure of the *N*^2^-dG-IQ adduct when positioned at position G^1^ of the *Nar*I sequence.

### Summary

Analysis of the *N*^2^-dG-IQ adduct placed at position G^3^ of the *Nar*I sequence (68–71,73,74), where it has been observed to cause −2 bp deletions when bypassed by hpol η (72), reveals that it adopts a base-displaced intercalated conformation in which the H4a and CH_3_ protons of the IQ ring face the minor groove and the H7a, H8a and H9a protons face the major groove. The IQ ring is shielded from water and stacks with the 5′- and 3′-neighbor base pairs. Remarkably, despite this conformational perturbation, the *N*^2^-dG-IQ adduct does not destabilize the duplex, which may correlate with the observation that it is refractory to repair by NER (63,99). In addition, the IQ moiety disrupts the potential for Watson–Crick hydrogen bonding with incoming dNTPs, which perhaps explains why this lesion blocks DNA synthesis by many polymerases.

## ACCESSION NUMBERS

The structural coordinates were deposited in the Protein Data Bank (www.rcsb.org): The PDB ID code for the *N*^2^-dG-IQ duplex is 2MAV.

## SUPPLEMENTARY DATA

Supplementary Data are available at NAR Online.

## FUNDING

National Institutes of Health (NIH) [R01 CA55678 to M.P.S., R01 ES016561 to C.J.R.]; Center [P30 ES000267, P30 CA068485]; instrumentation [S10 RR05805, S10 RR025677]; National Science Foundation Instrumentation [DBI 0922862] and the latter funded by the American Recovery and Reinvestment Act of 2009 (Public Law 111-5); Vanderbilt University assisted with the purchase of NMR instrumentation; K.M.S. and E.K.H. acknowledge support from National Institutes of Health predoctoral traineeship [T32 ES007028]. Funding for open access charge: NIH.

*Conflict of interest statement.* None declared.

## Supplementary Material

Supplementary Data
