# Supplementary material for: Base-displaced intercalation of the 2-amino-3-methylimidazo[4,5-f]quinolone N2-dG adduct in the NarI DNA recognition sequence
Source: Nucleic Acids Res. 2013 Dec 22;42(5):3450–63. doi: 10.1093/nar/gkt1109 (PMC3950664; doi:10.1093/nar/gkt1109)
Supplement: Supplementary Data [file supp_42_5_3450__index.html]

Base-displaced intercalation of the 2-amino-3-methylimidazo[4,5-f]quinolone N2-dG adduct in the NarI DNA recognition sequence — Base-displaced intercalation of the 2-amino-3-methylimidazo[4,5-f]quinolone N2-dG adduct in the NarI DNA recognition sequence — Supplementary Data 

# Base-displaced intercalation of the 2-amino-3-methylimidazo[4,5-*f*]quinolone *N*2-dG adduct in the *Nar*I DNA recognition sequence

## Supplementary Data

files

**Files in this Data Supplement:**

- Supplementary Data - pdf file
