# Supplementary material for: Base-displaced intercalation of the 2-amino-3-methylimidazo[4,5-f]quinolone N2-dG adduct in the NarI DNA recognition sequence
Source: Nucleic Acids Res. 2013 Dec 22;42(5):3450–63. doi: 10.1093/nar/gkt1109 (PMC3950664; doi:10.1093/nar/gkt1109)

Base-Displaced Intercalation of the 2-Amino-3-methylimidazo[4,5-*f*]quinolone N^2-dG Adduct in the *Nar*I DNA Recognition Sequence

Supporting Data

Kallie M. Stavros[1], Edward K. Hawkins[1,2], Carmelo J. Rizzo[1] and Michael P. Stone[1,*]

[1] Department of Chemistry, Center in Molecular Toxicology, Vanderbilt-Ingram Cancer Center, and Vanderbilt Institute of Chemical Biology, Vanderbilt University, Nashville, Tennessee, 37235-1822, United States of America
[2] Deceased

*To whom correspondence should be addressed. Tel: 1-615-322-2589; Email: michael.p.stone@vanderbilt.edu

Table S1. Non-exchangeable Proton Chemical Shift Table for N^2-dG Modified Duplex (ppm).

Nucleotide	H1'	H2'	H2"	H3'	H8/H6	H5/H2
C^1	5.72	2.10	2.43	4.49	7.73	5.78
T^2	5.96	2.39	2.05	4.49	7.45	1.51
C^3	5.45	2.21	1.86	4.64	7.25	5.44
G^4	5.40	2.56	2.52	4.85	7.67	
G^5	5.70	2.35	2.51	4.82	7.55	
C^6	5.78	1.48	2.21	4.64	6.85	4.98
X^7	5.96	2.65	2.45	4.88	8.04	
C^8	5.48	2.23	1.76	4.41	7.40	5.05
C^9	5.20	2.23	1.95	4.66	7.21	5.31
A^{10}	6.11	2.56	2.77	4.87	8.13	7.52
T^{11}	5.84	2.29	1.87	4.88	7.00	1.32
C^{12}	6.09	2.06	2.09	4.41	7.40	5.58
G^{13}	5.47	2.56	2.41	4.67	7.70	
A^{14}	6.12	2.77	2.57	4.88	8.11	7.74
T^{15}	5.52	2.17	1.75	4.64	6.87	1.14
G^{16}	5.42	2.50	2.55	4.85	7.59	
G^{17}	5.76	2.37	2.45	4.94	7.46	
C^{18}	6.29	2.61	2.11	4.88	7.98	6.08
G^{19}	5.28	2.05	2.42	4.42	7.18	
C^{20}	5.72	2.23	1.87	4.64	7.04	4.93
C^{21}	5.26	2.11	1.76	4.63	7.15	5.35
G^{22}	5.27	2.58	2.53	4.83	7.70	
A^{23}	5.97	2.76	2.51	4.87	7.91	7.62
G^{24}	5.83	2.08	2.22	4.45	7.44	

Table S2. Exchangeable Proton Chemical Shift Table for N^2-dG IQ Modified Duplex (ppm).

Base Pair	N1H/N3H	H4a	H4b
C^1:G^{24}	13.37	7.21	7.79
T^2:A^{23}	14.02		
C^3:G^{22}	12.90	7.04	8.61
G^4:C^{21}	13.05	6.81	8.53
G^5:C^{20}	12.89	6.29	8.14
C^6:G^{19}	12.52	6.66	8.30
X^7:C^{18}	11.56		
C^8:G^{17}	11.59	6.05	7.89
C^9:G^{16}	12.58	6.68	8.30
A^{10}:T^{15}	13.52		
T^{11}:A^{14}	13.72		
C^{12}:G^{13}	12.61	7.11	8.19

Figure S1. Thermal melting profiles of the N^2-dG IQ modified duplex and the corresponding unmodified duplex. The graph shows the first derivative plots of UV absorbance measured at 260 nm as a function of temperature.

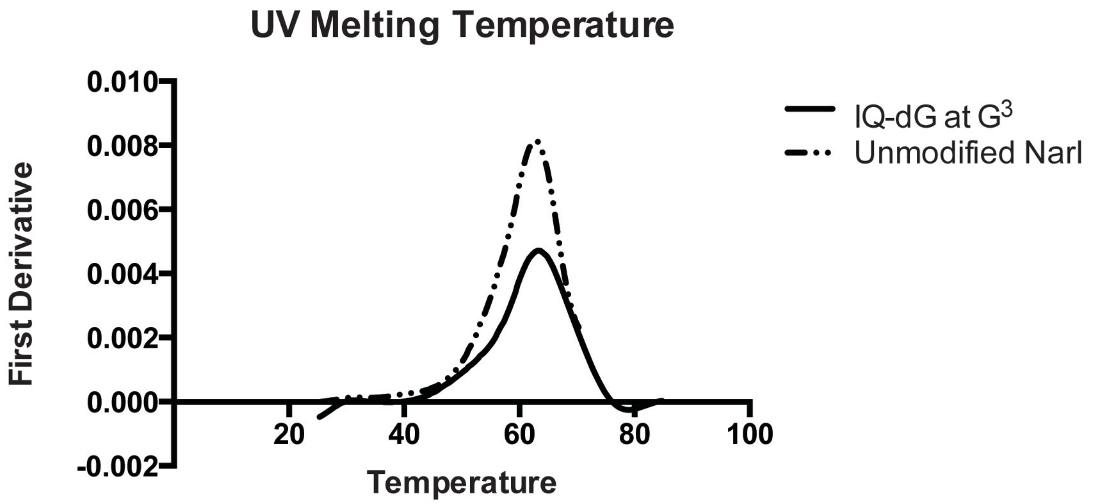

Figure S2. Partial charges calculated for the N^2-dG IQ adduct and used for potential energy minimization and rMD calculations.

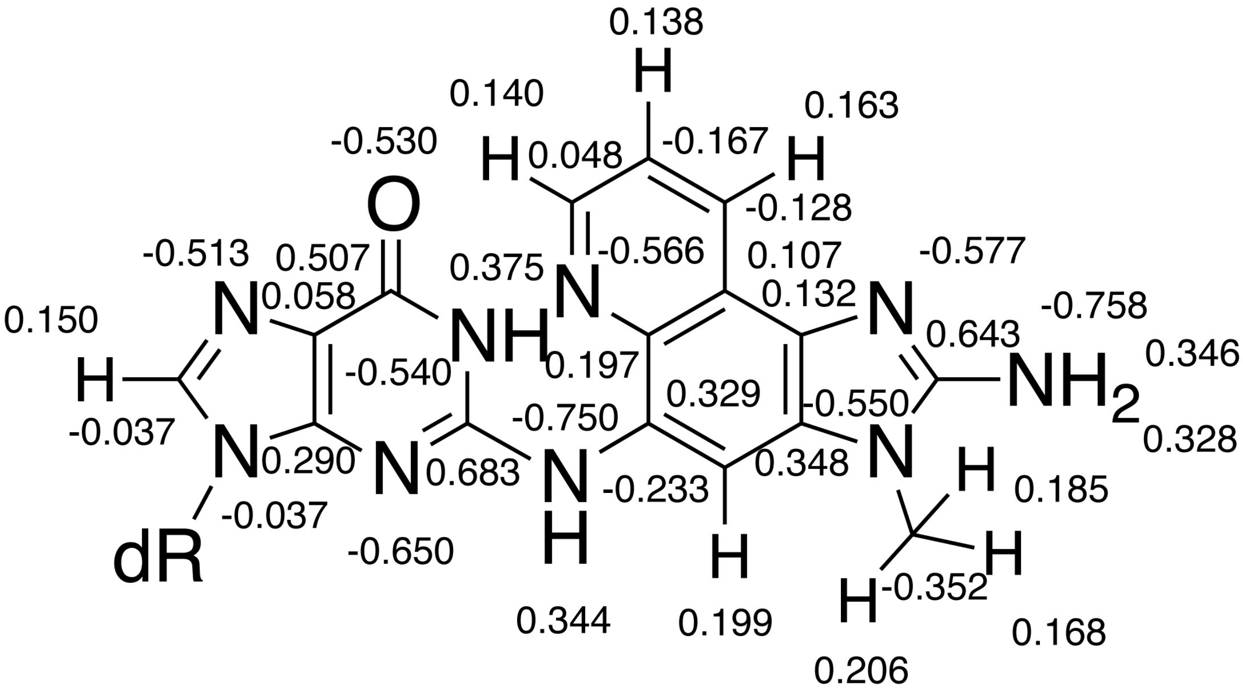

Supplement: Supplementary Data [file supp_gkt1109_nar-02804-f-2013-File002.pdf]
